# Revolutionizing bladder tumor treatment with non-thermal atmospheric plasma treatment under local anesthesia: a case report

**DOI:** 10.1093/jscr/rjaf863

**Published:** 2025-10-31

**Authors:** Dolev Perez, David Dothan, Boris Chertin

**Affiliations:** Department of Urology, Shaare Zedek Medical Center, PO B 3235, Jerusalem 91031, Israel; Faculty of Medicine, The Hebrew University, Jerusalem, Israel; Department of Urology, Shaare Zedek Medical Center, PO B 3235, Jerusalem 91031, Israel; Faculty of Medicine, The Hebrew University, Jerusalem, Israel; Department of Urology, Shaare Zedek Medical Center, PO B 3235, Jerusalem 91031, Israel; Faculty of Medicine, The Hebrew University, Jerusalem, Israel

**Keywords:** bladder cancer, NMIBC, non-thermal atmospheric plasma, TURBT, tumor

## Abstract

Non-muscle-invasive bladder cancer exhibits low progression risk with high recurrence rate. Standard resection requires general anesthesia and carries inherent risks. CAPS Medical™ PlasmaSure™ is an innovative system that employs non-thermal atmospheric plasma in a minimally invasive procedure, and a first-in-human study has previously demonstrated its ability to safely and efficiently ablate superficial bladder tumors. The current case report shows that the same procedure can be conducted without the need for general anesthesia. These findings highlight a future potential for PlasmaSure™ to enable in-office efficient ablation of superficial bladder tumors, thereby minimizing anesthesia-related risks and enhancing patient quality of life.

## Introduction

Most urothelial bladder tumors are low-grade (LG) and non-invasive and thus pose a low risk of progression. However, they have a high rate of recurrence, which places a significant burden on patients and the healthcare system [1–3]. The standard of care involves transurethral resection of the bladder tumor (TURBT) conducted under general or spinal anesthesia in the operating room. However, TURBT is associated with risks of morbidity, bleeding, and scarring, which can negatively impact bladder function and patient health-related quality of life [4–6]. Furthermore, TURBT often requires extended hospital stays, with a median of two days, and some patients require up to 8 days of hospitalization and involves general or regional anesthesia [7]. This is also the case for small tumors, which constitute the majority of recurrent cases. Thus, there is a clear demand for minimally-invasive, highly selective procedures performed in the doctor office, thereby eliminating the need for general anesthesia and hospitalization. The PlasmaSure™ system by CAPS Medical™ may offer this solution.

Non-thermal atmospheric plasma, also known as non-thermal plasma (NTP), is a stream of ionized noble gases (e.g. helium or argon) produced at near-room temperature and atmospheric pressure [8]. NTP selectively induce a cytotoxic insult in malignant cells, which eventually leads to their death by apoptosis, without causing thermal or other clinically meaningful damage to the healthy tissue around the tumor [9, 10].

**Figure 1:**
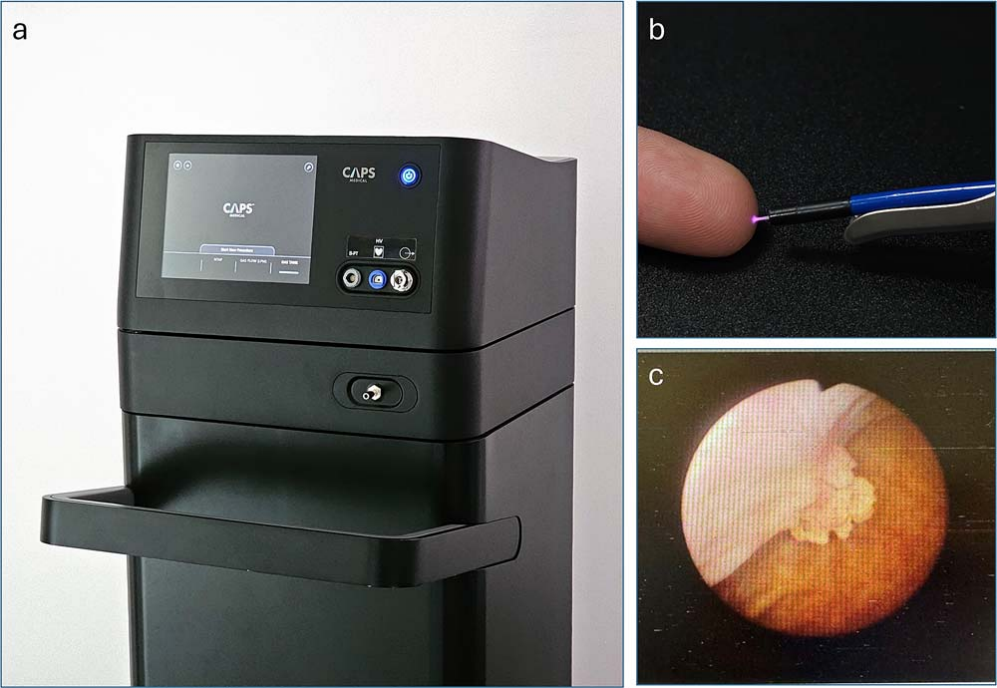
(a) The PlasmaSure™ console, operated using a touchscreen interface. It generates and monitors the PlasmaSure™ parameters and gas flow during the clinical procedure. (b) The PlasmaSure™ probe applied through a working channel of the 22 Fr cystoscope. (c) A cystoscopy image of the treated tumor at baseline.

The PlasmaSure™ system (Fig. 1a) is an investigational device developed and manufactured by CAPS Medical Ltd. and is designed to treat a wide range of tumors and lesions. The system is comprised of a PlasmaSure™ generator and a single-use, disposable PlasmaSure™ probe, which is small enough to enable treatment endoscopically utilizing existing endoscopic tools. The small-diameter, disposable, flexible probe is introduced through a working channel of minimally invasive tools (such as cystoscopes) and delivers NTP directly to the tumor. The dosage of NTP delivered to the tissue and the gas pressure within the bladder are controlled in real-time. This innovative technology shows promise as an in-office alternative to traditional procedures (e.g. TURBT, fulguration, and laser ablation) that overcomes many of their limitations.

The PlasmaSure™ system supports various probes designed for either flexible or rigid cystoscopy, while using the same generator and delivering the same plasma output. In this study, the setup employed flexible probes designed for use via rigid cystoscopy in the controlled environment of the OR (Fig. 1b).

Herein, we describe the first reported case of a bladder tumor treated with PlasmaSure™ technology under local anesthesia, which paves the way for convenient, in-office procedures.

## Case presentation

We present a case of a 68-year-old woman with a history of TaLG bladder cancer. A recent abdominal ultrasound showed a small polypoid mass near the right orifice within the urinary bladder. Subsequent flexible cystoscopy confirmed the presence of a 1 cm papillary tumor near the right orifice (Fig. 1c).

The patient was otherwise healthy, and the physical examinations showed no abnormalities. The computed tomography urography and urine cytology results were negative for upper tract pathology or high-grade disease.

The patient provided informed consent and subsequently underwent tumor ablation under local anesthesia using the PlasmaSure™ system investigational device. Throughout the procedure, the patient’s vital signs, including oxygen saturation, blood pressure, electrocardiogram, and heart rate, were continuously monitored. Intravenous gentamicin was administered for antibiotic prophylaxis. After sterile preparation, the patient was placed in the standard lithotomy position. A 22 Fr rigid cystoscope was inserted transurethrally, and the bladder was insufflated with helium gas from the PlasmaSure™ system to enhance visualization and distend the bladder. The PlasmaSure™ system maintained a constant pressure of helium gas at 12 mmHg throughout the procedure. Following a visual examination of the bladder, the PlasmaSure™ probe was positioned at the target lesion, and PlasmaSure™ treatment was delivered for 6 min. The patient tolerated the procedure well under local anesthesia and did not require any additional sedation. No complications or adverse events occurred, and the patient was discharged 1 hr after the procedure, required no pain medication, and resumed normal daily activities the same day. As per the clinical trial protocol, the patient returned for a follow-up visit 3 weeks after the procedure. Cystoscopy revealed a complete response, with a full disappearance of the treated tumor. The bladder mucosa appeared normal, with no evidence of scarring or ulceration. No tumor recurrence occurred during the 13-month follow-up.

## Discussion

The management of LG, stage Ta (NMIBC) presents a persistent challenge for both urologists and patients, primarily because of frequent tumor recurrence.

The standard TURBT procedure is performed under general or spinal anesthesia, which is nowadays considered safe and reliable, with advancements in anesthetic techniques having significantly minimized risks [11]. These risks are further increased in patients with specific risk factors, such as advanced age, a history of smoking, and pre-existing comorbidities, which are frequently observed in bladder cancer patients [12, 13]. Therefore, the potential risks associated with general anesthesia must be carefully weighed against the benefits of the planned procedure, especially in patients with these identified risk factors. Conversely, local anesthesia has a profoundly lower risk and requires less postoperative nursing care, minimal postoperative pain relief, and shorter recovery time, thereby resulting in substantially lower treatment costs [14].

Several authors support the use of local anesthesia as an effective alternative to the traditional procedure of bladder tumor resection using lasers or fulguration [15–17]. However, the use of the aforementioned techniques is limited, as the American Urological Association guidelines for non-invasive bladder cancer recommend the use of fulguration only in cases of sub-centimeter, and recurrent papillary tumors and do not address the majority of patients [18]. This case study demonstrates the successful treatment of a bladder tumor using PlasmaSure™ under local anesthesia. While this procedure was performed using rigid cystoscope, the procedure was well-tolerated, had no complications, and resulted in favorable oncological outcomes.

## Conclusion

Our data suggest that the PlasmaSure™ system might be used with flexible cystoscopy for safe and effective in-office ablation of bladder tumors without anesthesia.
